# Genetic Structure of *Plasmodium falciparum* and Elimination of Malaria, Comoros Archipelago

**DOI:** 10.3201/eid1611.100694

**Published:** 2010-11

**Authors:** Stanislas Rebaudet, Hervé Bogreau, Rahamatou Silaï, Jean-François Lepère, Lionel Bertaux, Bruno Pradines, Jean Delmont, Philippe Gautret, Philippe Parola, Christophe Rogier

**Affiliations:** Author affiliations: Institute for Biomedical Research of the French Army, Marseille, France (S. Rebaudet, H. Bogreau, L. Bertaux, B. Pradines, C. Rogier);; Université de la Méditerranée, Marseille (J. Delmont, P. Parola);; Assistance Publique–Hôpitaux de Marseille, Marseille (P. Gautret);; Programme National de Lutte contre le Paludisme, Moroni, Comoros (R. Silaï);; Dispensaire de Bandraboua, Mayotte, France (J.-F. Lepère)

**Keywords:** *Plasmodium falciparum*, malaria, elimination, control, population genetics, molecular epidemiology, parasitic diseases, drug resistance, Comoros, research

## Abstract

The efficacy of malaria control and elimination on islands may depend on the intensity of new parasite inflow. On the Comoros archipelago, where falciparum malaria remains a major public health problem because of spread of drug resistance and insufficient malaria control, recent interventions for malaria elimination were planned on Moheli, 1 of 4 islands in the Comoros archipelago. To assess the relevance of such a local strategy, we performed a population genetics analysis by using multilocus microsatellite and resistance genotyping of *Plasmodium falciparum* sampled from each island of the archipelago. We found a contrasted population genetic structure explained by geographic isolation, human migration, malaria transmission, and drug selective pressure. Our findings suggest that malaria elimination interventions should be implemented simultaneously on the entire archipelago rather than restricted to 1 island and demonstrate the necessity for specific chemoresistance surveillance on each of the 4 Comorian islands.

## Abstract 2

Elimination interventions should be implemented simultaneously throughout the entire archipelago.

*Plasmodium falciparum* causes malaria worldwide; 250 million cases and ≈1 million deaths occur annually, mostly in sub-Saharan Africa. However, recently increased international financial commitment has revived hope for malaria elimination from selected areas to which it is endemic, and the feasibility of elimination has become a topic of research (*1*). The successful elimination of malaria from several Caribbean islands, Cyprus, Reunion, Mauritius, Maldives, Taiwan, and Singapore in the context of the Global Malaria Eradication Program (1955–1968) (*2*) suggests that islands are prime targets for elimination interventions. Because most parasites among neighboring areas are exchanged through human migrations, the geographic isolation of islands can limit malaria importation and may make control easier (*3*,*4*).

Several molecular epidemiologic studies have shown that *P. falciparum* populations are more or less homogeneous within malaria-endemic areas and may exhibit genetic structure patterns shaped by various transmission rates and geographic isolation levels (*4*–*6*). Although geographic isolation may be more relevant on islands than within continents, the role of parasite genetic structure in malaria-endemic archipelagos or among malaria-endemic islands and the nearest continent remains unknown. Past failures of malaria elimination in Zanzibar off the coast of mainland Tanzania; in Sri Lanka (*1*); or in Mayotte, a France-administered island of the Comoros archipelago (*7*), raise the question of the minimal geographic isolation level and the optimal size of intervention area required for malaria elimination success. Analysis of malaria epidemiology in Comoros archipelago, where a limited malaria elimination program is ongoing, may help to answer this question.

Falciparum malaria remains a major public health problem on the 4 islands of the Comoros archipelago (Grande Comore, Moheli, Anjouan, and Mayotte) (Table 1) in the Indian Ocean between Madagascar and the eastern coast of Africa. Malaria control has been hampered by the emergence of *P. falciparum* resistance to chloroquine and to pyrimethamine/sulfadoxine in the early 1980s (*7*,*10*) and of *Anopheles* mosquitoes resistance to DDT. Malaria control also has had recurrent political, economic, and structural weaknesses in the Union of the Comoros (the state comprising Grande Comore, Moheli, and Anjouan islands). Under stable political and economic conditions, notable efforts in case management and vector control in Mayotte failed to eliminate falciparum malaria and to prevent recurrent epidemics (Table 1). During the past 6 years Since 2004, health authorities in Grande Comore and France have introduced an artemisinin-based combined therapy (artemether plus lumefantrin) as first-line treatment for uncomplicated falciparum malaria (*7*,*10*). Large-scale distribution of insecticide-treated mosquito nets also has been gradually implemented on Grande Comore, Moheli, and Anjouan (*8*), with the goal of reaching up to 89.1% and 46.3% of the households with at least 1 mosquito net and 1 insecticide-impregnated mosquito net, respectively, among 1,620 households from the 3 islands (Comoran National Malaria Control Program, unpub. data, 2007).

**Table 1:** Epidemiologic and sampling characteristics of 5 sites studied for *Plasmodium falciparum* malaria, Comoros archipelago and Marseille, France

Characteristic	Grande Comore	Moheli	Anjouan	Mayotte	Marseille
Area, km^2^	1,148	290	424	374	
Total population	330,000	40,000	280,000	190,000	50,000–80,000
No. bites by infected mosquitoes/person*	10–20/y (up to 200/y)	10/y (up to 1/night)	No data	Low	None
Endemicity*†	Mesoendemic to hyperendemic	Mesoendemic to hyperendemic	Mesoendemic to hyperendemic	Hypoendemic	None
Total no. reported cases (% confirmed cases), 2006*	51,148 (34)	7,866 (27)	15,408 (19)	496 (100)	84 (100)
Incidence/1,000 inhabitants, 2006*	150	150	50	3	Null
% *P. falciparum* malaria cases,* 2006	96	96	96	90	97
Period of sampling, 2007	Apr–May	Apr–May	Apr–May	Entire year	Entire year
No. patients sampled, 2007	62	61	63	227	111
Median age of sampled patients, y (IQR) ‡	4 (2–14.8)	7.5 (2.6–21)	7 (3.3–18)	19 (15–25)	33 (9.8–40)
No. sampled patients in site (A) with history of recent arrival from another site (B)	7	17	6	13	111§
Anjouan	6	9	–	7	
Grande Comore	–	8	5	6	
Mayotte	1	1	0	–	
Moheli	0	–	2	0	
No. randomly genotyped isolates	36	36	36	36	36

*Data from World Malaria Report 2008 (*8*) and from various Comorian and French official reports and references published in French, all reviewed by the first author in a recent, unpublished thesis (Rebaudet S. Molecular epidemiology and population genetics study of *Plasmodium falciparum* in Comoros archipelago. Impacts on malaria control [thesis] [in French]. Marseille (France): Université de la Méditerranée; 2009). Cases for Marseille represent those in persons with history of recent arrival from one of the 4 islands. †Malaria endemicity levels based on 2–9 years of available parasite prevalence data, according to the World Health Organization classification: hypoendemic, 0–10%; mesoendemic, 11%–50%; hyperendemic, 51%–75%; and holoendemic, >75% (*9*). ‡IQR, interquartile range. §Mainly from Grande Comore (S. Rebaudet, pers. comm.).

In Mayotte, anti–*Anopheles* spp. mosquito larvae measures have been strengthened. Finally, by late 2007, a controversial malaria elimination project was launched on the sole island of Moheli with assistance from China. Mass treatment of the residing and disembarking population with artemisinin plus piperaquine (Artequick; Artepharm Co., Guangzhou, People’s Republic of China) and primaquine was initiated without enhancement of vector control. Because of continual human travel across the archipelago, the long-term success of such a spatially limited elimination attempt is questionable.

In addition, surveillance of *P. falciparum* chemosusceptibility has been chaotic and unequal among the islands of the archipelago, and results of the few available therapeutic efficacy tests and in vitro and molecular resistance studies often have been discordant. A more rational and efficient surveillance system is urgently needed. Because Marseille, France, houses a Comorian community of 50,000–80,000 persons who annually import several hundred malaria cases, the city was proposed as a relevant surveillance site for chemosusceptibility of *P. falciparum* imported from Comoros (*11*). However, extrapolating these chemoresistance data to the entire archipelago remains difficult.

As already proposed for Borneo (*12*) and the Philippines (*13*), our main objective was to analyze the genetic structure of *P. falciparum* on the Comoros islands to 1) forecast the chances of middle-term and long-term success for the current elimination program focalized in Moheli, 2) guide future malaria elimination programs on the archipelago, and 3) adjust its chemoresistance monitoring and treatment policies. Study results also would provide a pertinent model for determining which other malaria-endemic areas might be eligible for malaria elimination. A secondary objective was to assess whether the diversity of the *P. falciparum* strains imported into Marseille were representative of the *P. falciparum* populations from Comoros so we could evaluate the relevance of distant chemoresistance surveillance from Marseille.

We characterized *P. falciparum* populations from each of the 4 islands and from Marseille (imported from the archipelago) by multilocus microsatellite genotyping. The genetic polymorphism of 3 genes involved in *P. falciparum* resistance to chloroquine, pyrimethamine and cycloguanil, or sulfadoxine was also investigated.

## Materials and Methods

### *P. falciparum* Isolates

The study was conducted in 2007 (before the malaria elimination program was launched in Moheli) in each of the 4 islands of the Comoros archipelago and in Marseille. The protocol was approved by the ethics committee of the university hospitals of Marseille and by the Comorian Ministry of Health. Blood samples were obtained after informed consent from patients seeking care for symptomatic falciparum malaria at healthcare centers of the archipelago or at emergency departments of hospitals in Marseille.

Blood samples were absorbed onto Whatman FTA Elute absorbent filter paper in Grande Comore, Moheli, and Anjouan islands, on Whatman 903 Protein Saver filter paper (Whatman Inc., Florham Park, NJ, USA) in Mayotte, and collected into Vacutainer tubes (Becton Dickinson, Le Pont-De-Claix, France) in Marseille. All samples were frozen and kept at –20°C. After eliminating samples with missing data or the lowest parasitaemia levels (<0.01%), 36 isolates per site were randomly chosen for genotyping, a sample size considered adequate for the planned population genetics analyses.

### Collection of Epidemiologic Data

Patient's age, sex, history of travel across or outside the archipelago (during the past year for Grande Comore, Moheli, and Anjouan; during the past 3 weeks for Mayotte) and history of recent clinical malaria episodes and intake of antimalarial drugs (during the previous month) were collected by oral questioning. Distances between each island were measured by using Google Earth software.

### Genotyping Procedures

DNA was extracted from filter papers according to the manufacturer's recommendations (Whatman Inc.) and from whole blood from Vacutainer tubes by using the EZNA Blood DNA Kit (Biofidal, Vaulx-en-Velin, France). Next, the entire genome was amplified by using the Illustra GenomiPhi V2 DNA Amplification Kit (GE Healthcare, Little Chalfont, UK).

### Molecular Markers

Length polymorphism was analyzed for 6 complex and putatively neutral microsatellite loci previously described (*4*): *Pf2689*, *7A11*, *C4M79*, *Pf2802*, *TRAP*, and *C4M69* (Table A1). The studied chemoresistance markers were the K76 point mutation of the *P. falciparum* chloroquine resistance transporter (*Pfcrt*) gene (associated with *P. falciparum* resistance to chloroquine) (*14*); point mutations of *P. falciparum* dihydrofolate reductase (*Pfdhfr*) gene codons 108, 16, 51, 59, and 164 (associated with *P. falciparum* resistance to pyrimethamine and cycloguanil, i.e., proguanil metabolite) (*15*); and *P. falciparum* dihydropteroate synthase (*Pfdhps*) gene codons 436, 437, 540, 581, and 613 (associated with *P. falciparum* resistance to sulfadoxine) (*15*) (Table A1).

### Genotyping by PCR

Microsatellite loci were amplified by nested PCR with fluorescent end-labeled primers before electrophoresis on polyacrylamide gels with Genescan-500 LIZ labeled size standards on an ABI 3130XL capillary sequencer (Applied Biosystems, Warrington, UK) (Table A1). Their length was then analyzed by using GENESCAN software (Applied Biosystems, Carlsbad, CA, USA), as described (*4*). The *Pfcrt* gene was amplified by seminested PCR, and the codon 76 mutation was genotyped by using a simple PCR-restriction fragment digest assay and fluorescent detection of products on an ABI 3130XL capillary sequencer, as described (*16*). The *Pfdhps* and *Pfdhfr* genes were amplified by nested PCR, and their mutations were genotyped by using a primer extension method, as described (*17*) and electrophoresis on the ABI 3130XL capillary sequencer.

### Statistical Analysis

The multiplicity of infection (MOI, i.e., the number of parasites genetically distinguishable by different alleles) with *P. falciparum* was estimated for each isolate from the microsatellite locus that exhibited the highest number of alleles. The mean MOI for each *P. falciparum* population (Grande Comore, Moheli, Anjouan, Mayotte, and Marseille) was then calculated. Each pair of sites was compared for MOI by using the Mann-Whitney U test.

For parasites with multiple infection, i.e., >1 allele at each locus, we conducted separate subsequent analysis considering the following: 1) complete dataset, 2) curtailed dataset with single or main clones after elimination of isolates unsuccessfully genotyped at all 6 microsatellite loci, or 3) reconstructed multilocus genotypes after elimination of samples with impossible reconstruction (>1 allele at >1 locus with equivocal peak intensities) and elimination of unsuccessfully genotyped isolates (Table 1).

### Genetic Diversity

Genetic diversity of the 5 *P. falciparum* populations was assessed by the number of alleles per locus and by the Nei unbiased expected heterozygosity index (*H_e_*) calculated from allelic frequencies on the 6 microsatellites for complete datasets by using GENETIX software version 4.05 (*18*,*19*). Comparison between *H_e_* of the 5 different populations was performed on FSTAT software version 2.9.4 with a 1,000 permutations bilateral comparison test (*20*).

### Population Genetic Structure

Population genetic structure was investigated by using the Wright F statistic (*F_ST_*) (*21*). The *F_ST_* index was computed for the 6 microsatellite markers and 5 populations on FSTAT software version 2.9.4 (*20*,*22*) and by using the Slatkin index on ARLEQUIN software (*23*). A canonical correspondence analysis of the reconstructed multilocus genotypes set was conducted to illustrate measures of population structure (*24*) by using CANOCO software (*25*), and its graphic representation was performed by using R software. A Monte Carlo procedure permuting genotypes among the populations was used to test the significance of the canonical axes and estimate the 95% confidence intervals of the centroid of each population (*25*).

### Frequency of Mutations Associated with Chemoresistance

We estimated the frequency of point mutations on the *Pfcrt* (K76T mutation), *Pfdhfr* (108 + 59 + 51 triple mutation), and *Pfdhps* (437 + 540 double mutation) genes. Differences among sites were tested by using the Fisher exact test.

### Associations between *F_ST_* and Estimations of Parasite Flux

The association between genetic distance (transformed as *F_ST_*/[1 – *F_ST_*]) and the natural log of the geographic distance in kilometers was investigated for each pair of islands according to the isolation-by-distance model (*5*,*12*,*26*). When we considered the number of patients in each sampled island (A) with history of recent arrival from each of the neighboring islands (B) and thus possibly with imported malaria (Table 1), the relationship between *F_ST_* and the mean proportion of these travelers among patients, calculated as ([N_B→A_/N_A_] + [N_A→B_/N_B_])/2, was investigated for each pair of islands.

## Results

*P. falciparum* was detected by PCR in each of the 36 genotyped blood samples from all 5 sites. Microsatellite genotyping was complete for 149 (83%) of the 180 samples (Table 2).

**Table 2:** Genotyping results of the 5 sites studied for *Plasmodium falciparum* malaria, Comoros archipelago and Marseille, France

Characteristic	Grande Comore	Moheli	Anjouan	Mayotte	Marseille	Total
No. randomly genotyped isolates	36	36	36	36	36	180
No. detected parasites	50	53	76	44	58	281
No. single or main clones successfully genotyped	28	20	29	36	36	149
No. reconstructed multilocus genotypes	30	24	40	37	39	170
No. multi-infected isolates	9	10	22	4	14	59

### Mean MOIs

Of the 180 samples, 59 isolates were multi-infected; the proportion of multi-infection among islands differed substantially (Table 2). The mean MOI ranged from 1.22 in Mayotte to 2.11 in Anjouan (Table 3). It was significantly higher in Anjouan than in Grande Comore (p = 0.0015), Moheli (p = 0.0051), and Mayotte (p = 0.0001) and higher in Marseille than in Mayotte (p = 0.0093).

**Table 3:** MOIs of *Plasmodium falciparum* infections for the 5 sites studied, Comoros archipelago and Marseille, France*

Locus	Grande Comore			Moheli			Anjouan			Mayotte			Marseille	
No.	MOI	No.	MOI	No.	MOI	No.	MOI	No.	MOI
All 6 loci	36	1.39		36	1.47		36	2.11		36	1.22		36	1.64
*Pf2689*	34	1.03		27	1.07		36	1.19		36	1.08		36	1.22
*7A11*	34	1.12		32	1.19		35	1.37		36	1.14		36	1.39
*C4M79*	34	1.21		25	1.32		33	1.55		36	1.11		36	1.42
*Pf2802*	31	1.00		23	1.00		32	1.00		36	1.00		36	1.00
*TRAP*	35	1.42		29	1.14		36	1.67		36	1.08		36	1.19
*C4M69*	32	1.06		21	1.19		34	1.12		36	1.03		36	1.11
Mean no. alleles per locus	8.5			8			8.5			4.5			7.8	

*Based on no. multi-infected isolates as shown in Table 2. MOI, multiplicity of infection (no. multi-infected isolates/no. randomly genotyped isolates); no., no. randomly genotyped isolates.

### Genetic Diversity

Genetic diversity (*H_e_*) of each population, estimated by unbiased expected heterozygosity based on allelic frequencies of the 6 microsatellites and the complete dataset, is shown in Table 4. The highest diversity was observed for Anjouan and Moheli (each *H_e_* = 0.71) and the lowest diversity for Mayotte (*H_e_* = 0.63). The mean *H_e_* was significantly lower for Mayotte than for Marseille (p = 0.04) and lower than for the other sites combined (p = 0.001).

**Table 4:** Genetic diversity of *Plasmodium falciparum* at the 5 sites studied, Comoros archipelago and Marseille, France*

Locus	Grande Comore			Moheli			Anjouan			Mayotte			Marseille	
	No.	*H_e_*		No.	*H_e_*		No.	*H_e_*		No.	*H_e_*		No.	*H_e_*
All 6 loci	50	0.63		53	0.71		76	0.71		44	0.51		58	0.63
*Pf2689*	35	0.47		29	0.44		43	0.52		39	0.15		42	0.53
*7A11*	38	0.81		38	0.85		48	0.82		41	0.41		50	0.87
*C4M79*	41	0.86		33	0.85		51	0.83		40	0.74		51	0.87
*Pf2802*	31	0.00		23	0.48		32	0.43		36	0.48		36	0.20
*TRAP*	39	0.79		33	0.80		57	0.85		39	0.60		43	0.56
*C4M69*	34	0.87		25	0.82		38	0.84		37	0.68		40	0.77

*No., no. detected parasites. *H_e_*, Nei unbiased expected heterozygosity index.

### Genetic Differentiation among Islands and Population Structure

**Figure 1:**
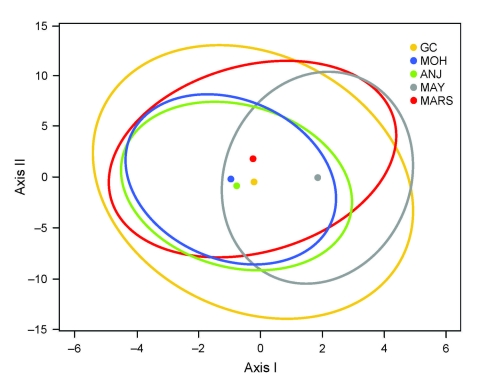
Results of canonical correspondence analysis (CCA) of *Plasmodium falciparum* populations from the islands of Grande Comore (GC), Moheli (MOH), Anjouan (ANJ), and Mayotte (MAY) and from Marseille, France (MARS), according to 6 microsatellite loci. CCA is used as a 2-dimensional representation of genetic distance between plasmodial populations assessed from 6 microsatellite loci (*Pf2689, C4M79, Pf2802, 7A11, TRAP*, and *C4M69*). This representation requires the projection of data from 6-dimensional space to 2-dimensional space. Canonical axes I and II of the new 2-dimensional space are calculated to conserve the highest genetic variance between populations after projection of data, and their significance was tested by Monte Carlo permutation that also enabled estimation of the 95% confidence intervals (ellipses) of the centroid (dots) of each population.

Figure 1 shows the centroid of each falciparum population surrounded by its 95% confidence interval, and both axes were significant (p = 0.0001 and p = 0.0004 for 1,000 permutations, respectively). Grande Comore, Moheli, and Anjouan nearby centroids suggest closely related populations. The detached Mayotte centroid suggests a marked differentiation from all the other populations.

**Figure 2:**
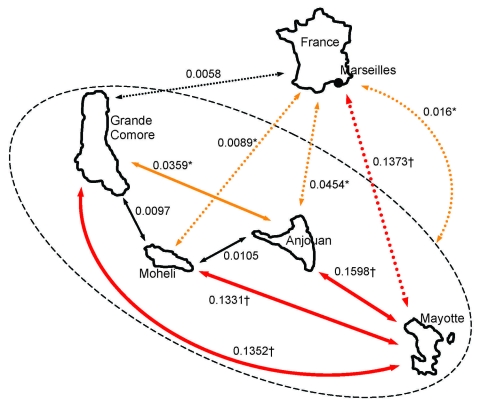
Genetic differentiation (*Fst*) between *Plasmodium falciparum* populations from the islands of Grande Comore (GC), Moheli (MOH), Anjouan (ANJ), and Mayotte (MAY) and from Marseille, France (MARS), according to 6 microsatellite loci. Pairwise comparison among sites that used complete dataset (n = 281) and 6 microsatellite loci (*Pf2689*, *C4M79*, *Pf2802*, *7A11*, *TRAP*, and *C4M69*). Departure of *F_ST_* from 0 tested after 10,000 bootstrap simulations and by using Bonferroni corrected p values obtained after 200 permutations. Difference is significant if adjusted p<0.005. Black arrows indicate negligible (*F_ST_*<0.01) and nonsignificant differentiation. Asterisks (*) and orange arrows indicate moderate (0.01<*F_ST_*<0.1) and/or statistically significant differentiation. Daggers (†) and red arrows indicate important (*F_ST_* >0.1) and significant differentiation. Plain arrows indicate genetic differentiation between the parasite populations of the Comoros islands. Dotted arrows indicate genetic differentiation between the parasite population imported in Marseille (from Comoros) and either the overall parasite population of the entire Comoros archipelago (dotted oval and extreme right arrow) or the parasite populations of each of the 4 islands.

Figure 2 shows the pairwise differentiation coefficients (*F_ST_*) estimated for the 5 parasite populations according to the 6 microsatellite loci and the complete dataset (n = 281). The number of *P. falciparum* clones used to calculate *F_ST_* between sites was 50, 53, 76, 44, and 58 in Grande Comore, Moheli, Anjouan, Mayotte, and Marseille, respectively. The Moheli parasite population did not differ significantly from the Grande Comore and Anjouan populations. Conversely, the Mayotte population differed significantly from the populations of the 4 other sites. Marseille parasite populations differed significantly from those from all sites except Grande Comore. Similar differentiation index were obtained by using a curtailed dataset (n = 149) or reconstructed multilocus genotypes (n = 170) and by using the Slatkin index (data not shown).

### Relations between *F_ST_* and Estimations of Parasite Flux

**Figure 3:**
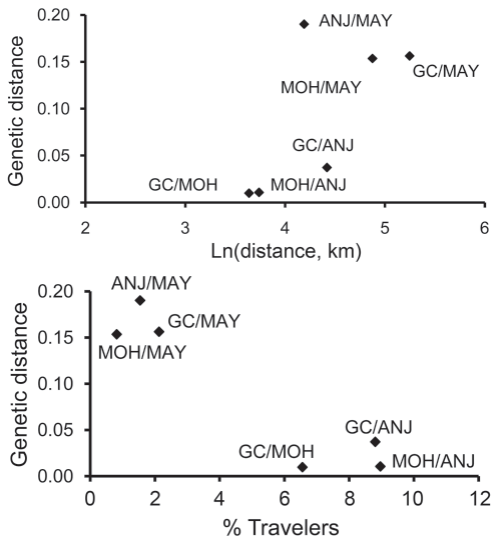
Relationship between geographic and genetic distances for each pair of Comoros islands (top) and between mean percentage of travelers among sampled patients and genetic distance for each pair of Comoros islands (bottom). Genetic distances were calculated as (*F_ST_*/1 – *F_ST_* ), where *F_ST_* is the Wright F statistic. Mean percentage of travelers was calculated from the total number of sampled patients in one site (N_A_) with history of recent arrival from another site (N_B_) by using the equation ([N_B→A_/N_A_] + [N_A→B_/N_B_])/2; data in Table 1. GC, Grande Comore; MOH, Moheli; ANJ, Anjouan; MAY, Mayotte; Ln, logarithmically transformed.

Association between genetic and geographic distances for each pair of islands is shown in Figure 3. No association was significant. The Anjouan–Mayotte pair exhibited a large *F_ST_* despite the close proximity of the 2 islands.

Of the 414 patients sampled in the archipelago, 35 reported recent travel to neighboring islands (Table 1). Figure 3 suggests a negative relationship between the mean percentage of travelers among patients and the corresponding *F_ST_*.

### Frequency of Point Mutations associated with Chemoresistance

Prevalence of *Pfcrt*, *Pfdhfr*, and *Pfdhps* mutations in the 5 *P. falciparum* study populations are presented in Table 5. Prevalence of the *Pfcrt* mutation (i.e., isolates with the 76T allele or with the 76 K, and T alleles) was significantly lower in Anjouan than in the other parasite populations (p<0.002). Prevalence of the *Pfcrt* mutation was significantly higher in Mayotte than in any other population (p< 0.0001). The prevalences of the *Pfcrt* mutation in Grande Comore, Moheli, and Marseille did not differ significantly.

**Table 5:** Frequency of chemoresistance-associated point mutations of 5 sites studied for *Plasmodium falciparum* malaria, Comoros archipelago and Marseille, France*

Locus	No. isolates (% mutations)				
	Grande Comore	Moheli	Anjouan	Mayotte	Marseille
*Pfcrt*					
76T	33 (45.5)	33 (45.5)	36 (13.9)	36 (91.7)	36 (52.8)
76 T and K	33 (9.1)	33 (6.1)	36 (5.6)	36 (2.8)	36 (0)
K76 (Wt)	33 (45.5)	33 (48.5)	36 (80.6)	36 (5.6)	36 (47.2)
*Pfdhfr*					
108N	26 (50.0)	19 (84.2)	34 (38.2)	36 (50.0)	36 (80.6)
108 N and S	26 (15.4)	19 (5.3)	34 (11.8)	36 (0)	36 (2.8)
S108 (Wt)	26 (34.6)	19 (10.5)	34 (50.0)	36 (50.0)	36 (16.7)
59R	26 (50.0)	19 (78.9)	34 (26.5)	36 (44.4)	36 (77.8)
59 R and C	26 (7.7)	19 (10.5)	34 (11.8)	36 (0)	36 (2.8)
C59 (Wt)	26 (42.3)	19 (10.5)	34 (61.8)	36 (55.6)	36 (19.4)
51I	26 (38.5)	19 (63.2)	34 (23.5)	36 (44.4)	35 (65.7)
51 I and N	26 (11.5)	19 (5.3)	34 (2.9)	36 (0)	35 (2.9)
N51 (Wt)	26 (50.0)	19 (31.6)	34 (73.5)	36 (55.6)	35 (31.4)
108N and 59R	26 (57.7)	19 (89.5)	34 (38.2)	36 (44.4)	36 (80.6)
108N and 59R and 51I	26 (50.0)	19 (68.4)	34 (26.5)	36 (44.4)	35 (68.6)
*Pfdhps*					
437G	25 (4.0)	24 (20.8)	31 (0)	36 (0)	36 (8.3)
437 G and A	25 (0)	24 (4.2)	31 (0)	36 (0)	36 (5.6)
A437 (Wt)	25 (96.0)	24 (75.0)	31 (100.0)	36 (100.0)	36 (86.1)
540E	25 (0)	24 (4.2)	31 (0)	36 (0)	36 (0)
540 E and K	25 (4.0)	24 (0)	31 (0)	36 (0)	36 (0)
K540 (Wt)	25 (96.0)	24 (95.8)	31 (100.0)	36 (100.0)	36 (100.0)
437G and 540E	25 (0)	24 (0)	31 (0)	36 (0)	36 (0)

*Pfcrt, P. falciparum chloroquine resistance transporter; *Pfdhpr*, *P. falciparum* dihydrofolate reductase; *Pfdhps*, *P. falciparum* dihydropteroate synthase.

When mutated, the *Pfdhfr* gene frequently exhibited the association of the 3 mutations 108N + 59R + 51I. Prevalence of this triple mutation was significantly lower in the Anjouan population than in the Grande Comore (p = 0.04), Moheli (p = 0.003), or Marseille populations (p = 0.0004). Its prevalence also was significantly higher in Marseille than in Mayotte (p = 0.02). The prevalence of *Pfdhps* gene mutations appeared low in the 5 populations.

Multi-infected isolates with genotype ambiguities and impossible distinction between associated clones were rare. However, prevalence of these mutations varied little, regardless whether these ambiguous multi-infected isolates were considered.

## Discussion

The mean MOIs remained low for the Comoros archipelago in comparison with African areas, where malaria is highly endemic (*4*), most likely because of moderate levels of malaria transmission (*4*,*5*). Likewise, the significantly higher MOI in Anjouan may reflect a higher level of malaria transmission in the rainy and swampy sampled areas, where vector control has for a long time been impaired by recurrent island-specific political crises. The genetic diversities appeared lower on the archipelago than on most of the African continent (*4*,*5*,*27*–*30*), probably because of the geographic isolation of the islands and their lower malaria transmission levels that could limit effective parasite population sizes and outbreeding. However, genetic diversities remained higher than in Asia (*5*,*6*,*12*,*13*) and South America (*5*,*31*).

The genetic differentiation index (*F_ST_*) exhibited a contrasted genetic structure between the studied *P. falciparum* populations. Genetic distances were low among parasites on Grande Comore, Moheli, and Anjouan islands. However, *F_ST_*s among these 3 populations and Mayotte were as significant as between *P. falciparum* populations of Senegal and Djibouti when the same microsatellite loci were used (*4*) or as between populations of Africa and Southeast Asia when other microsatellite loci were used (*5*). In addition, the genetic distances between falciparum populations across the archipelago seemed associated with the parasite flows among islands, estimated from the proportion of travelers among sampled patients, in particular for Moheli, Grande Comore, and Anjouan.

Our results strongly suggest that despite the insular geographic isolation of Moheli and the malaria elimination program launched in late 2007 on this island only, the mass treatment without enhanced vector control may soon be impaired by the continuous importation of new parasites through intense human migrations. In addition to flights and ferries regularly traveling across the archipelago, humans in Comoros move mainly by small fishing boats, especially from and toward Moheli (S. Rebaudet, pers. comm.). Their ubiquitous and informal traffic makes human flux estimations unreliable (probably several tens of thousands of persons each way annually [S. Rebaudet, pers. comm.]) and their control difficult. These factors might explain why, despite the substantial resources that France has allocated in Mayotte to malaria control since the mid-1970s, malaria importation to this island could not be stopped and autochthonous falciparum malaria could not be eliminated. The disease persists in Mayotte with a hypoendemo-epidemic setting, genetically characterized by low MOI, low *H_e_*, significant linkage disequilibrium (data not shown), and high *F_ST_*s, artificially overestimated by the sampling of multiple repeated genotypes (data not shown).

The persistent efforts for malaria elimination in Moheli can be hypothesized to create a Mayotte-like setting requiring efficient vector control to prevent epidemics in a Mohelian population that is losing its immunity. In the Union of the Comoros, the extension of the elimination program based on artemisinin-based combined therapy mass treatment to Grande Comore and Anjouan is being considered by Comorian health authorities and their Chinese interlocutors (R. Silaï, pers. comm.). Its success and the prevention of epidemics will depend on the rapid and large implementation of the preventive, diagnostic, and therapeutic measures planned with the funds granted in 2010 by Round 8 of the Global Fund (http://portfolio.theglobalfund.org/Grant/Index/COM-810-G03-M?lang=en).

Isolation of a specific *P. falciparum* population before planning its elimination needs to be appropriately evaluated. Results from the present Comorian epidemiologic study illustrate how it could be evaluated by a population genetics approach. In that type of geographic setting, population genetics studies provide a probably more direct and reliable estimation of parasite flows and risk for re-introduction than does the evaluation of human population movements by sociodemographic methods. Therefore, the relevance of parasite inflow from Africa (mostly the Tanzania coast, Madagascar, or other malaria-endemic areas) should be evaluated before the elimination project is extended to the rest of the Comoros archipelago. Similar data would also be useful for Sri Lanka, Malaysia, Indonesia, the Philippines, Solomon islands, or Vanuatu, several islands where national or localized malaria elimination projects are being implemented (*2*).

According to the genetic structure of *P. falciparum* populations in Comoros demonstrated by microsatellite genotyping, resistance levels would be expected to be fairly similar across the archipelago, except for Mayotte. However, the study of *Pfcrt* and *Pfdhfr* resistance–associated mutations differed markedly, explainable only by contrasting levels of drug selective pressure among islands. Indeed, the prevalence of the K76T mutation on the *Pfcrt* gene was high in both Grande Comore and Moheli as found in previous studies (*32*,*33*) but substantively lower in Anjouan and significantly higher in Mayotte where chloroquine use was massive during 1975–2007 (*7*,*34*). Similarly, the prevalence of *Pfdhfr* triple mutants was higher in Moheli than in Anjouan and the prevalence of *Pfdhfr* double or triple mutants higher in Marseille than in Grande Comore.

Although no reliable estimation of past use of antimalarial drugs in Comoros is available, these differences may be explained by a greater use in Moheli of pyrimethamine (in the sulfadoxine/pyrimethamine combination for malaria treatment) and trimethoprim (in cotrimoxazole compound, which is widely prescribed in this island as an antimicrobial drug) and in Marseille of proguanil (in association with chloroquine or atovaquone, used as malaria chemoprophylaxis by travelers to the archipelago) (S. Rebaudet, pers. comm.). Trimethoprim and proguanil are 2 antifolate drugs whose cross-resistance with pyrimethamine has been suspected (*35*,*36*) and that may have selected these *Pfdhfr* mutations. Because of the contrasting resistance levels among islands, the risk for rapid propagation of resistant *P. falciparum* strains across the archipelago suggested by the low *F_ST_*s among Grande Comore, Moheli, and Anjouan (*4*,*5*), and the easier selection of multigenic resistance and multiresistance from low MOIs limiting the possibilities of genetic recombinations that could break apart allele combinations (*5*,*37*,*38*), French and Comorian health authorities should organize surveillance of chemoresistance, both regular and separated for each island.

Finally, microsatellite genotypes of the *P. falciparum* population in Marseille substantially differed from those populations on all islands except Grande Comore. Because most of the Comorian inhabitants living in Marseille originated from Grande Comore, malaria is imported mainly from this particular island (S. Rebaudet, pers. comm.). Therefore, if we consider that the *P. falciparum* population in Marseille may be representative only of the Grande Comore population and the distinct levels of drug pressure between Marseille and the other populations, the relevance of distant chemosusceptibility surveillance from Marseille is likely to be limited.

## Appendix

**Table A1:** Primer sequences and amplification conditions of the 6 microsatellite loci and *Pfcrt*, *Pfdhfr*, and *Pfdhps* genes of *Plasmodium falciparum**

Loci	GenBank accession no.	Size, bp	Hr	Primer ID	Primer sequences for first and second rounds of nested PCR (5′ → 3′)	Ta, °C
Pf2689	G37854	86	2	Pf2689 PCR1	TTA ACC TTA TAG CTT CAG AG	56
					TCT TCT TCA CTT ACA TTA AAG	
				Pf2689 PCR2	TAT GCA CAC ACG TTT CTA	54
					6-FAM—CTC CAA GGC ATT CAC GTA	
7A11	G38831	92	7	7A11 PCR1	ACA TAT TAT TTC TTC GTA A	53
					TTA TCT CTT CTC TGA GTA A	
				7A11 PCR2	ATG TGT AAG GAG ATA GTA TA	54
					6-FAM—CAA CTT TCT CTT TTT AAA TAT TAC	
C4M79	G42726	220	3	C4M69 PCR1	TTT TGT AGG AAC ATG TAA	53
					GGA GAC TAG CTC TAC AAT A	
				C4M69 PCR2	TTT ATA TCA AGA ATG ACA ACC	57
					NED—TAG CAA CAA TAA ACA ATA TGG	
Pf2802	G37818	136	5	Pf2802 PCR1	GAT GCT TAG TTT AAT CTT ATA ACA AAT A	60
					GAC TTA CTT TCT TAC ATA AAA TCA TTA AC	
				Pf2802 PCR2	GTA TAA AAG GAA ATA CCT A	52
					NED—CAG ACT ATC TTA AGG GAA	
TRAP	G37858	134	3	TRAP PCR1	ATA AAA CAA ATT ACC GAG TA	56
					ACA ATT CAG ATT ACC TGA A	
				TRAP PCR2	CAT AAT AGT AGC AAG AGA	49
					PET—GAT TAT ATA TAG CGA TTT AC	
C4M69	G37956	362	4	C4M69 PCR1	AGA AAT GGA GAT AAA CTA TTA CAA CTA	60
					AGC GCA CGA GAA CAA TC	
				C4M69 PCR2	GAA ATG GAG ATA AAC TAT TAC	61
					VIC—AAT TAC ACA ACA GAT GTG AA	
Pfcrt			7	Pfcrt PCR1	GTT CTT GTC TTG GTA AAT GT	50 then 45
					CCA ATT TTG TTT AAA GTT CT	
				Pfcrt PCR2	GTT CTT GTC TTG GTA AAT GT	50 then 45
					6-FAM—TAA ATG TGC TCA TGT GTTTA	
Pfdhfr			4	Pfdhfr primers (template)		
				dhfr PCR1	TTC TCC TTT TTA TGA TGG AAC AAG T	56
					ATA TTT GAA AAT CAT TTG GAT GTA TAG	
				dhfr PCR2	ACG TTT TCG ATA TTT ATG C	47
					TCA CAT TCA TAT GTA CTA TTT ATT C	
				Pfdhfr primers (SNaPshot)		
				dhfr51-f	AGG AGT ATT ACC ATG GAA ATG TA	
				dhfr16-r	gactgactCTC ATT TTT GCT TTC A AC CTT ACA ACA T	
				dhfr108-f	gactgactACA AAA TGT TGT AGT TAT GGG AAG AAC AA	
				dhfr164-r	ctgactgactgactgactAAT TCT TGA TAA ACA ACG GAA CCT CCT A	
				dhfr59-r	ctgactgactgactgactgactTGA TTC ATT CAC ATA TGT TGT AAC TGC AC	
Pfdhps			8	Pfdhps primers (template)		
				dhps PCR1	GATTCTTTTTCAGATGGAGG	52
					TTCCTCATGTAATTCATCTGA	
				dhps PCR2	GTT GAA CCT AAA CGT GCT GT	49
					TTC ATC ATG TAA TTT TTG TTG TG	
				Pfdhps primers (SNaPshot)		
				dhps613-r	TTG ATC ATT CAT GCA ATG GG	
				dhps540-f	gactGAG GAA ATC CAC ATA CAA TGG AT	
				dhps581-r	TAA GAG TTT AAT AGA TTG ATC ATG TTT CTT C	
				dhps436(1)-r	gactgactAGT GTT ATA GAT ATA GGT GGA GAA TCC	
				dhps436(2)-r	gactgactgactgactTGG ATT AGG TAT AAC AAA AAG GAI CA	
				dhps437-r	gactgactgactgactgactTTT TTG GAT TAG GTA TAA CAA AAG GA	

*Size in basepairs for 3D7 clone. *Pfcrt*, *P. falciparum* chloroquine resistance transporter; *Pfdhpr*, *P. falciparum* dihydrofolate reductase; *Pfdhps*, *P. falciparum* dihydropteroate synthase; Chr, chromosome. Primer sequences are given for reactions no. 1 (first round) and no. 2 (second round) of the nested PCRs and are 5′ → 3′ with fluorescent label (VIC, NED, 6-FAM or PET) and annealing temperature (Ta, °C). Thermocycling performed in a Biometra (Goettingen, Germany) 96-well T3 thermocycler.
